# Supplementary material for: Novel Driver Strength Index highlights important cancer genes in TCGA PanCanAtlas patients
Source: PeerJ. 2022 Aug 11;10:e13860. doi: 10.7717/peerj.13860 (PMC9375969; doi:10.7717/peerj.13860)
Supplement: Supplemental Information 2 [file peerj-10-13860-s002.zip › COHORTS/genes plots/cohorts/2021_8_16_14_9_distribution_cohorts_females_THCA.pdf]

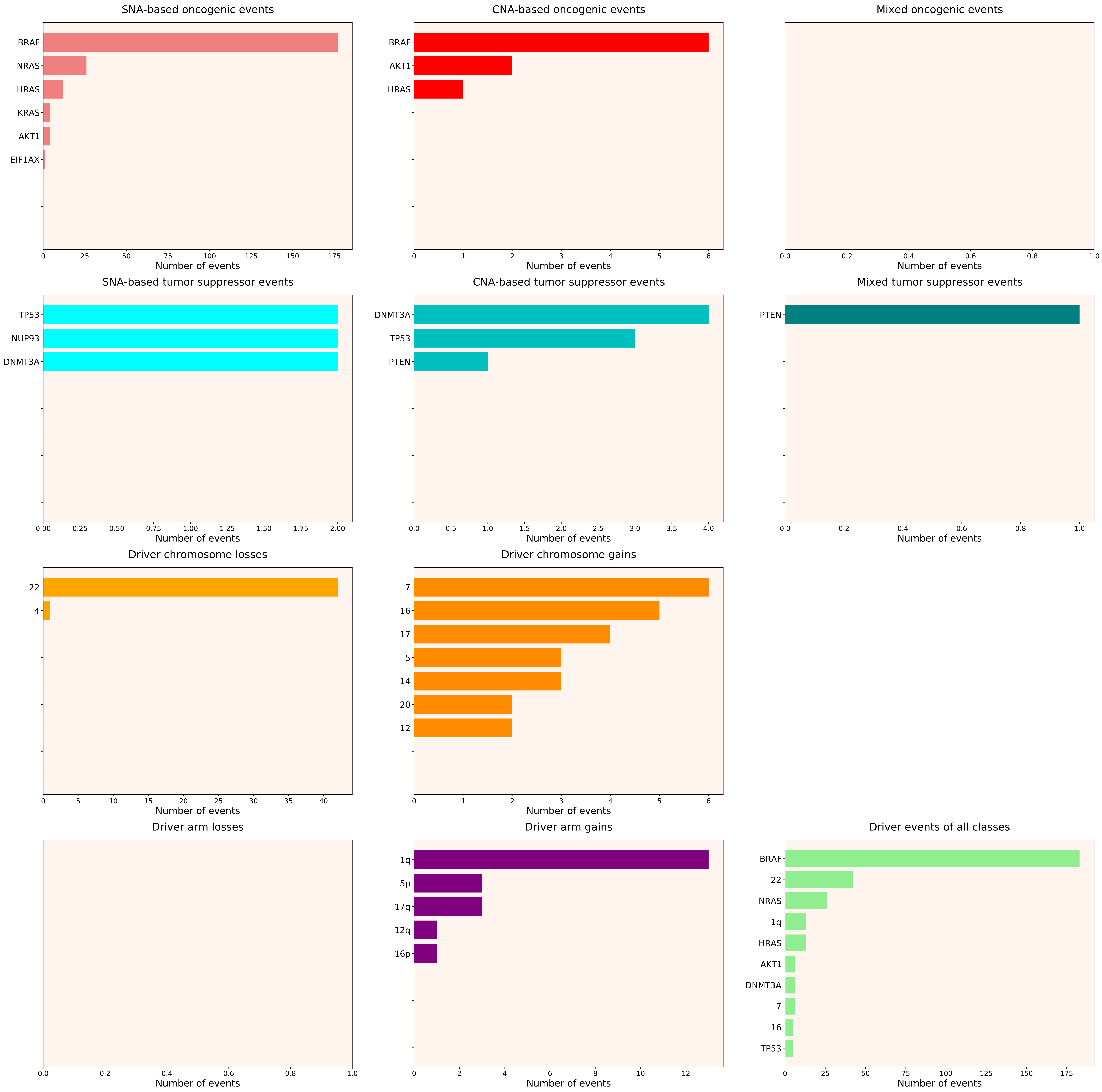

SNA-based oncogenic events
BRAF
NRAS
HRAS
KRAS
AKT1
EIF1AX
0 25 50 75 100 125 150 175
Number of events
CNA-based oncogenic events
BRAF
AKT1
HRAS
0 1 2 3 4 5 6
Number of events
Mixed oncogenic events
0.0 0.2 0.4 0.6 0.8 1.0
Number of events
SNA-based tumor suppressor events
TP53
NUP93
DNMT3A
0.00 0.25 0.50 0.75 1.00 1.25 1.50 1.75 2.00
Number of events
CNA-based tumor suppressor events
DNMT3A
TP53
PTEN
0.0 0.5 1.0 1.5 2.0 2.5 3.0 3.5 4.0
Number of events
Mixed tumor suppressor events
PTEN
0.0 0.2 0.4 0.6 0.8 1.0
Number of events
Driver chromosome losses
22
4
0 5 10 15 20 25 30 35 40
Number of events
Driver chromosome gains
7
16
17
5
14
20
12
0 1 2 3 4 5 6
Number of events
Driver arm losses
0.0 0.2 0.4 0.6 0.8 1.0
Number of events
Driver arm gains
1q
5p
17q
12q
16p
0 2 4 6 8 10 12
Number of events
Driver events of all classes
BRAF
22
NRAS
1q
HRAS
AKT1
DNMT3A
7
16
TP53
0 25 50 75 100 125 150 175
Number of events
